# Students’ Attitudes to and Knowledge of Brown Bears (*Ursus arctos* L.): Can More Knowledge Reduce Fear and Assist in Conservation Efforts?

**DOI:** 10.3390/ani11071958

**Published:** 2021-06-30

**Authors:** Vesna Oražem, Tadeja Smolej, Iztok Tomažič

**Affiliations:** Department of Biology, Biotechnical Faculty, University of Ljubljana, Večna Pot 111, 1001 Ljubljana, Slovenia; vesna.orazem7@gmail.com (V.O.); tadeja.smolej@gmail.com (T.S.)

**Keywords:** brown bear (*Ursus arctos*), attitudes, knowledge, workshops, conservation

## Abstract

**Simple Summary:**

Large carnivores distributed throughout Europe have interactions with people because their habitats often collide with human settlements. Since human behavior can significantly influence the conservation of these species, knowledge of certain behaviors and factors of influence are crucial. The present study included 534 students from lower and upper secondary schools. In this article, students’ attitudes to and knowledge of brown bears and the indirect effect of teaching are examined. Factors such as gender and seeing a bear in nature were found to influence the students’ attitudes and knowledge significantly, implying that they should be considered in any future educational actions. Other factors like residence, owning a dog, having a hunter in the family, breeding livestock and visiting a zoo had a smaller effect on the students’ attitudes and knowledge in general. The results thus indicate that greater knowledge was correlated with proconservation attitudes and reduced fear among the students.

**Abstract:**

The expansion of large carnivores across Europe is posing a challenge to their conservation. Since success with conservation may depend significantly on human behavior, knowledge of certain behaviors’ emergence and all the factors that affect them are crucial. The present study included 534 students who were divided into a comparison group (*n* = 317) and a treatment group (*n* = 217) consisting of 309 lower secondary (LS, M_Age_ = 12.2, SD = 0.94) and 225 upper secondary (US, *n* = 225, M_Age_ = 16.5, SD = 0.99) school students. We assessed their attitudes to and knowledge of brown bears. An indirect effect of the workshops (instructions) is also described. Sociodemographic factors, such as gender and seeing a bear in nature, significantly influenced the students’ attitudes and knowledge. Residence, owning a dog, having a hunter in the family, breeding livestock and visiting a zoo had a smaller effect on the students’ attitudes and knowledge. The results thus show that greater knowledge is correlated with proconservation attitudes, and partly with reduction of fear. Therefore, future conservation and management should employ strong communication, especially education activities based on direct experiences and carefully designed information regarding species and socio-scientific issues.

## 1. Introduction

The expansion of large carnivore species across Europe [1] adds to the challenges entailed in conserving them [2]. Brown bears (*Ursus arctos* L.) in Slovenia belong to the northern part of the wider Dinaric-Pindos population, spreading spatially and in abundant numbers [3]. The latest genetic estimation of the size of the brown bear population in Slovenia was made in 2015. The minimum yearly estimate for that year was 599 (545–655) individuals [4]. The most recent population estimate was made in 2018 and based on mathematical modeling. For that year, the maximum yearly abundance was 975 (875–1130) bears [5]. Across Europe, large carnivores such as brown bears are spreading because of increasing forest cover, crop coverage, shrinking human population density and forest fragmentation. These species have considerable adaptability, which means that large their coexistence with humans depends not simply on habitat suitability, which is already present, but rather on humans’ acceptance and policies [6]. In the majority of Slovenia, bear habitats border on human settlements, making interactions with people inevitable. Even if the population is considered stable, human-bear conflicts are still the biggest threat to the successful long-term conservation of the species [3].

Since human behavior can significantly impact conservation success, knowledge of the emergence of certain behaviors, and all the factors that affect them, are crucial [7]. The conservation of carnivore species is subject to sociopolitical and biological factors [8]. Therefore, all conservation and management actions should also include understanding people’s attitudes toward large carnivores [9]. In such actions, attitudes are often believed to change behaviors directly, yet the link is far more complex. In the theory of planned behavior [10] and the theory of reasoned action [11,12,13], the “behavioral intention” or intention to act is defined as a key precursor of the individual’s behavior. Personal attitudes and “subjective norms” are determinants of these acting intentions. Attitudes generally have three main components: affective, cognitive and conative [14]. Knowledge has been stated as the “structural property of attitudes” [15]. Several studies, e.g., [16,17] linked more knowledge with greater consistency in attitudes and behavior. Some authors e.g., [18] pointed to the importance of the individual knowledge dimension for attitudes, not just the amount of knowledge itself. After a person has acquired sufficient knowledge and skills, adequate decisions regarding behavior can be made [19]. On the other hand, the importance of feelings and emotions should not be neglected. When predicting management options in the case of brown bears and wolves, feelings can play a more important role than cognitions and knowledge itself [20].

While exploring environmental attitudes, two main dimensions emerge: preservation and utilization [21,22]. This approach offers insight into a respondent’s support for or opposition to conservation or management actions, providing a valuable orientation for future activities. However, if we wish to influence attitude formation in the long term, especially among children and adolescents, it is crucial to research the factors that influence attitudes, especially knowledge and teaching approaches.

Due to their importance for species conservation, attitudes to large carnivores have been researched extensively. However, most studies considered the general public or stakeholder groups, yet only a handful of such studies focused on children and students’ attitudes, e.g., [23,24,25,26,27,28,29,30,31,32]. Some research into attitudes to brown bears, as well as knowledge of the species, has already been conducted in Slovenia [33,34,35], but none of it has concentrated on school students and the influence of teaching (e.g., workshops). In the present study, the authors aimed to fill the gap in a particular segment of the population because most of the studies considered the general public or stakeholder groups.

For that reason, lower secondary and upper secondary students’ attitudes to, and knowledge of, brown bears were assessed. Along with these two measured variables, the indirect effect of the workshops (instructions) is also shown.

Building on the above background, the following research questions (RQ) were explored in our research:

**RQ 1.** Do socio-demographic factors like place of living (rural, suburban, urban area; bear area, bear occurrence area, non bear area), gender, owning a dog, having a hunter in the family, breeding livestock, having encountered a bear in nature or visiting a zoo influence students’ attitudes to and knowledge of bears and bear topics?
**RQ 2.** Do students’ attitudes to and knowledge of bears and bear topics vary by education level (comparing lower and upper secondary school programs)?
**RQ 3.** Does the amount of knowledge influence individuals’ attitudes to this species?
**RQ 4.** Can the indirect influence of instructions (the workshops performed) be detected in the students’ attitude and knowledge?

## 2. Materials and Methods

### 2.1. Sample

First, schools from different regions were contacted and were offered workshops that were organized within the LIFE DINALP BEAR project. In addition, schoolteachers were asked if they would be willing to participate in an additional study where students’ attitudes and knowledge regarding bear topics would be assessed. Schools had the decision to participate in an additional study or not, which makes it very difficult to achieve higher sample sizes. Ten lower secondary schools and four upper secondary schools participated in the study. For those schools, we randomly assigned classrooms for treatment and control groups. Questionnaires were administered in one session either before or after the workshops took place and were administered by the two persons who were conducting workshops (see Study design). The initial sample consisted of 542 lower and upper secondary school students. Students who submitted incomplete questionnaires were excluded from the final sample (*n* = 8). The final sample, therefore, included 534 students, divided into a comparison (*n* = 317) and a treatment group (*n* = 217) consisting of 309 lower secondary (LS, M_Age_ = 12.2, SD = 0.94) and 225 upper secondary (US, *n* = 225, M_Age_ = 16.5, SD = 0.99) school students, of whom 45.9% were male (*n* = 245) and 53.0% female (*n* = 283). Six students did not report their gender (1.1%). The majority of the students lived in a rural area (54.3%). The remaining students lived in a suburban area (19.9%) or an urban area (25.8%). The research focus was on participants who live in areas of permanent bear presence, with 71.2% of the sample meeting this criterion. The other students lived in an area of either occasional bear presence (21.2%) or without the presence of bears (4.9%). Other factors, such as a hunter in the family, livestock breeding, owning a dog, visiting a zoo, or having encountered a bear in nature, were also recorded. Among the whole sample, just 20.8% of the students reported a family relationship with a hunter. Only 8.8% of students reported that their families breed livestock, and 62.4% that they owned a dog. Most students reported visiting a zoo less than once a year or never (64.6%). The other respondents visit a zoo more frequently (34.6%). Most students (63.7%) had also not seen a bear in nature before the research took place and were not included in a similar workshop (75.5%). The workshop and survey implementation took place in the 2016–2017 school year. Since the testing was done for educational purposes and participation was anonymous and voluntary, ethical approval was not needed in Slovenia. Before or during the survey (relevant for the comparison group) and intervention (relevant for the treatment group), no other lessons about large carnivores had been given to the students.

### 2.2. Teaching Materials Regarding Brown Bear (LIFE DINALP BEAR Project)

The teaching materials (teachers’ manual, worksheets, and a PPT presentation for immediate use) [36] were designed as part of the LIFE DINALP BEAR project and are compatible with existing science and biology curricula. The materials are also structured according to difficulty (educational) levels. The LIFE DINALP BEAR project’s main goal was to establish the transboundary conservation and management of brown bears, with species’ population-level monitoring, management and conservation (across the northern Dinaric Mountains and south-eastern Alps area), to reduce human-bear conflicts, to promote coexistence as well as the species’ natural expansion from the Dinaric Mountains into the Alps [37]. The workshops were conducted as teacher-centered presentations of bear systematics, ecology, genetics, and measures supporting human-bear coexistence, and as student-centered workshops where students worked with biological materials like a bear skull, footprints and fur. In the project, workshops were also organized for teachers to facilitate the long-term use of the prepared teaching materials in an in-school setting.

The effectiveness of designed workshops was measured indirectly. This approach was selected due to two-person research execution only, and other organizational constraints (i.e., regular classroom interruptions with our research).

### 2.3. Study Design

**Figure 1:**
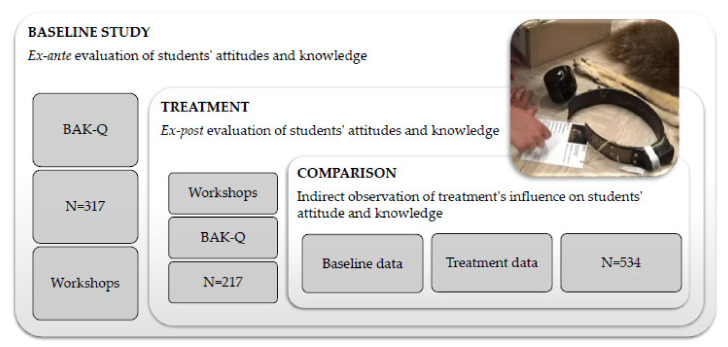
Study design.

The study was divided into three parts and two subsamples (in both, the participation of lower and upper secondary school students was evenly distributed) (see Figure 1):

**Study part 1.** The students’ (subsample 1: comparison group) attitudes to and knowledge of bears and topics related to the species (baseline study) were assessed using a Bears Attitude and Knowledge Questionnaire (BAK-Q). In this part, an ex ante evaluation of the students’ attitudes and knowledge was performed. After the attitudes and knowledge assessment, some students participated in the workshops (received the treatment) but were not included in the post workshop survey.
**Study part 2.** Students’ attitudes and knowledge were assessed in an ex post evaluation (subsample 2: treatment group). After the treatment, the students’ attitudes and knowledge were measured using the same BAK-Q.
**Study part 3.** Comparing the two groups: attitudinal ratings and knowledge scores of the comparison group (baseline data) and the treatment group (treatment data). Comparison of both subsamples entailed nonequivalent datasets.

To avoid the effect of the teacher as much as possible [38], the students were taught by two persons, one a prospective preservice biology teacher and the other an in-service biology teacher, both experienced in conducting similar workshops on large carnivores, (e.g., [28]) and in teaching in informal learning environments. The workshop consisted of teacher-centered instruction, a lecture on selected bear topics (bear systematics, ecology, genetics, coexistence with humans), and a student-centered workshop that encompassed three workstations at which various bear topics were presented. All workshops were incorporated into the regular science or biology classes.

### 2.4. Measure

A methodologically similar questionnaire, previously used for assessing attitudes and knowledge about wolves [27,28], was adopted to assess the students’ attitudes to, and knowledge about, bears (Appendix A). In the first part of the Bears attitude and Knowledge Questionnaire (BAK-Q), the students’ knowledge of bears was assessed based on true/false statements [26] related to bear biology, management, and conservation. To minimize the possibility of guessing, an “I Don’t know” option was offered for each question. The second part of the questionnaire contained 15 attitudinal items (statements) covering different attitudinal dimensions: (1) willingness to learn about bears; (2) acceptance (fear) of bears, and (3) views regarding the species’ conservation (with reference to Slovenia). For attitudinal items, a 5-point Likert scale was used. Similar items were used in prior studies where authors assessed participants’ attitudes to wolves (i.e., [27,28]) and/or other animal species or animal groups (i.e., [30,31,32,33,34,35,36,37,38,39,40,41,42]). The questionnaire’s third part gathered the respondents’ sociodemographic information.

### 2.5. Data Analysis

Principal component analysis (PCA) with an Oblimin rotation was used to explore whether selected attitudinal items fit within an individual attitudinal dimension (a principal component). For the final solution, only eigenvalues > 1 were considered. Bartlett’s test of sphericity and the Kaiser-Meyer-Olkin (KMO) measure of the sampling adequacy test were calculated to assess PCA appropriateness on the present dataset. Besides a critical KMO value of >0.70 [43], a minimum loading of at least 0.40 was used [44].

Extracted PCs (from the second, attitudinal part of the questionnaire) and summed knowledge scores (from the first, knowledge part of the questionnaire) were used as dependent variables and further analyzed according to independent variables such as gender, lower or upper secondary school (education level), place of residence (rural, suburban, urban; within an area of constant, occasional, or no bear presence), owning a dog, having a hunter in the family, livestock breeding, and having encountered a bear in nature. In the following General Linear Model (GLM) analysis, gender, education level, and place of residence were treated as fixed factors, and knowledge as a covariate. Other variables (having a hunter in the family, breeding livestock, owning a dog, and visiting a zoo) were excluded from GLM analysis due to the small sample sizes, but were applied when analyzing the effect of individual independent variable on students’ attitude and knowledge. The mean knowledge score was further used to categorize the students with regard to knowledge into low (mean score < M − 1SD), middle (mean score within M ± 1SD), and high student achievements (mean score > M + 1SD). The purpose of categorizing the knowledge scores was to compare the respondents’ attitudinal scores with their achievement. All statistical analyses were conducted using SPSS 20.0 software (SPSS, IBM Germany, Ehningen).

## 3. Results

The results are presented in four parts. First, the results of PCA and GLM are shown. The second part presents the baseline results of analyses of the students’ attitudes to and knowledge of bears (ex ante evaluation, using only the pretest), according to the selected independent variables. In the third part, ex post results of the students’ attitudes to, and knowledge of bears after the treatment (using the post workshop sample only) with respect to the selected independent variables are presented. The fourth part of the results describes the indirect impact of the treatment (the workshops) on the students’ knowledge.

### 3.1. Results of the Principal Component Analysis (PCA)

To explore whether the use of PCA was appropriate for the present dataset, the Kaiser-Meyer-Olkin (KMO) measure to test the sample’s adequacy and Bartlett’s test of sphericity were conducted. The results of both tests showed the PC structure was appropriate (see Table 1). Three meaningful PCs were extracted: (PC I) Conservation, (PC II) Interest to learn about bears, and (PC III) Fear: acceptance of bears.

### 3.2. Results of the General Linear Model (GLM): Multivariate Statistics

From Table 2 it can be seen that residence had no significant effect on the students’ attitude ratings. No interactions were found between the independent variables. The results show that the amount of knowledge regarding bears determined the students’ attitudes to this species the most. Other factors of significance were study level, gender, and direct experience of bears, while the place of residence did not affect the students’ attitude ratings.

### 3.3. Baseline Results of the Students’ Attitudes and Knowledge of Bears (Ex-Ante Evaluation)

**Figure 2:**
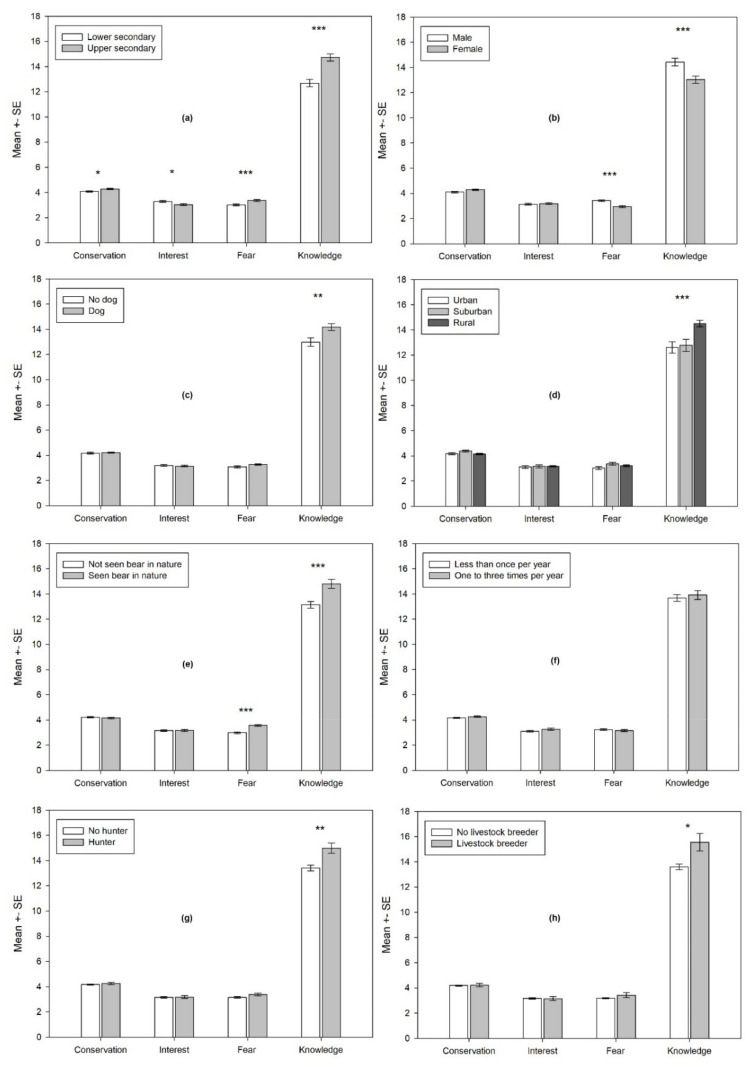
Differential effect of sociodemographic factors on the students’ attitudes and knowledge at the pretest: (**a**) Education level (lower or upper secondary school); (**b**) gender; (**c**) owning a dog; (**d**) place of residence; (**e**) seen a bear in nature; (**f**) visiting a zoo; (**g**) hunter in the family; (**h**) breeding livestock. Note: lower mean values on fear dimension mean more fear (lower acceptance). Note: * *p* < 0.05, ** *p* < 0.01, *** *p* < 0.001.

Lower secondary students (Figure 2a) expressed a slightly lower conservation attitude (Z = −2.142, *p* = 0.032), a somewhat greater interest to learn (Z = −2.412; *p* = 0.016), greater fear (Z = −3.443; *p* = 0.001), and significantly less knowledge (Z = −4.820; *p* < 0.001) of bears than upper secondary school students. Males (Figure 2b) showed far less fear (Z = −4.422; *p* < 0.001) and more knowledge (Z = −3.428; *p* = 0.001) than females. Students who are dog owners (Figure 2c) possessed more knowledge about bears (Z = −2.887; *p* = 0.004), while students who lived in rural areas (Figure 2d) knew more about bears than their counterparts in suburban and urban areas (χ^2^ = 17.101; df = 2; *p* < 0.001). Students who reported having seen a bear in nature (Figure 2e) expressed less fear (Z = −5.250; *p* < 0.001) and more knowledge about the species (Z = −3.343; *p* = 0.001). Visiting a zoo had no effect on the students’ attitudes or knowledge regarding bears (Figure 2f). Students whose family members or relatives are hunters (Figure 2g) expressed more knowledge about bears (Z = −3.021; *p* = 0.003), while those who come from livestock-breeding families (Figure 2h) also expressed slightly more knowledge (Z = −2.494; *p* = 0.013).

### 3.4. Ex Post Results of the Students’ Attitudes to and Knowledge of Bears after the Treatment

**Figure 3:**
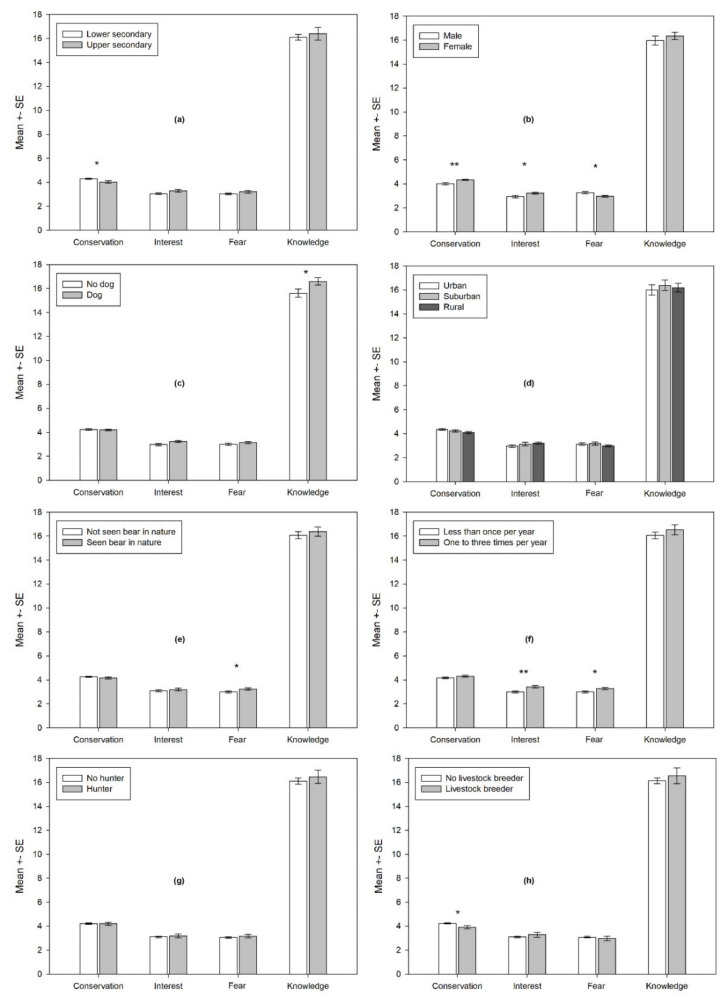
Differential effect of sociodemographic factors on the students’ attitudes and knowledge at the post-test: (**a**) education level (lower or upper secondary school); (**b**) gender; (**c**) owning a dog; (**d**) place of residence; (**e**) seen a bear in nature; (**f**) visiting a zoo; (**g**) hunter in the family; (**h**) breeding livestock. Note: lower mean values on fear dimension means more fear (lower acceptance). Note: * *p* < 0.05, ** *p* < 0.01.

Students from the lower secondary school (Figure 3a) expressed slightly higher conservation attitudes than their counterparts (Z = −2.098; *p* = 0.036). Females expressed higher proconservation attitudes (Z = −3.096; *p* = 0.002), showed somewhat greater interest to learn about bears (Z = −2.210; *p* = 0.027) and were slightly more afraid of them (Z = −2.322; *p* = 0.020) (Figure 3b). No gender-related differences were detected regarding what was known about bears. Dog owners (Figure 3c) expressed slightly more knowledge about bears (Z = −2.069; *p* = 0.039). No residence-related differences regarding attitudes and knowledge were identified (Figure 3d). Students who had already seen a bear in nature (Figure 3e) expressed slightly less fear (Z = −2.146; *p* = 0.032). Similarly, students who had visited a zoo more regularly (Figure 3f) reported less fear (Z = −2.225; *p* = 0.026). At the same time, they showed more interest to learn about bears (Z = −3.055; *p* = 0.002), yet they were not more knowledgeable about the topic. Having a hunter in the family had no influence on the students’ attitudes and knowledge (Figure 3g). Livestock breeding (Figure 2h) led to slightly lower proconservation attitudes (Z = −2.312; *p* = 0.021).

### 3.5. Indirect Impact of the Treatment (Workshops) on the Students’ Knowledge

**Figure 4:**
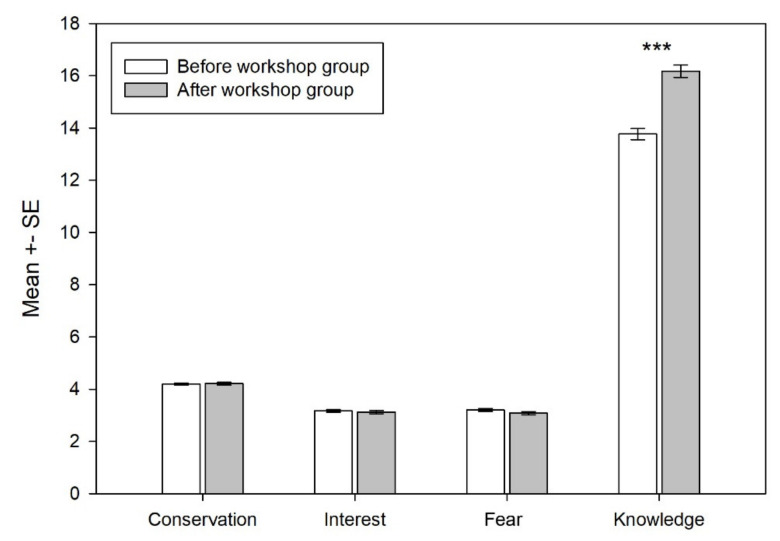
Effect of the instruction on the students’ attitudes to and knowledge of bears. Note: lower mean values on fear dimension means more fear (lower acceptance). Note: *** *p* < 0.001.

The after-workshop (treatment) group expressed significantly more knowledge about bears than the pre-workshop (comparison) group (Z = −5.019; *p* < 0.001). However, no statistically significant differences regarding their attitudes were detected (Figure 4). When putting knowledge scores into three categories (low, middle, and high scores), a significant difference between the comparison and treatment group was detected (χ^2^ = 26.235, df = 2, *p* < 0.001). There were 8.3% of low achieving students in the treatment group compared to 17.7% of low achieving students in the comparison group. The same applied for the numbers of high achieving students, where there were 24.4% high achieving students in treatment group compared to only 9.8% of high achieving students in comparison group.

**Figure 5:**
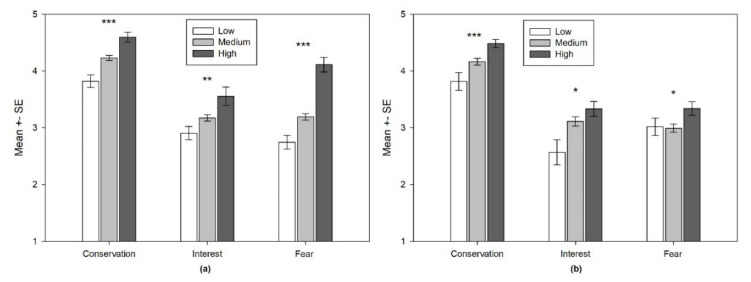
The effect of knowledge on different attitudinal dimensions: (**a**) the comparison group; **(b**) the treatment group; Note: lower mean values on fear dimension mean more fear (lower acceptance). Note: * *p* < 0.05, ** *p* < 0.01, *** *p* < 0.001.

The two groups expressed very similar attitudes. However, students (from both groups) who revealed more knowledge about bears than their counterparts also expressed significantly higher proconservation attitudes, greater interest to learn about bears and less fear of them (Figure 5). In the comparison group (Figure 5a), statistically significant differences were found between the knowledge categories and all the attitudinal dimensions: conservation (Kruskal-Wallis: χ^2^ = 26.096; df = 2; *p* = < 0.001), interest to learn (Kruskal-Wallis: χ^2^ = 10.698; df = 2; *p* = 0.005) and fear-acceptance (Kruskal-Wallis: χ^2^ = 40.283; df = 2; *p* < 0.001). Similar results were established for the treatment group (Figure 4b). A statistically significant effect of knowledge influenced all three attitudinal dimensions, among which the biggest difference between knowledge categories was seen in the conservation dimension (Kruskal-Wallis: χ^2^ = 14.800; df = 2; *p* = 0.001), followed by an interest to learn (Kruskal-Wallis: χ^2^ = 7.580; df = 2; *p* = 0.023) and the fear-acceptance dimension (Kruskal-Wallis: χ^2^ = 7.051; df = 2; *p* = 0.029). While conservation attitudes and an interest to learn were similar in the two groups, some differences were determined between the treatment and comparison groups in the “Fear-acceptance” dimension. Namely, in the comparison group, less knowledge corresponded with greater fear. However, these differences were partially eliminated within the treatment group (Figure 5b), where fear reduction among the students with the highest knowledge scores was less evident.

### 3.6. Correlations between Attitudinal Dimensions and Knowledge Scores

The correlations between attitudinal dimensions and attitudes regarding the knowledge scores were assessed for both the comparison (Table 3) and treatment groups (Table 4). In both groups, conservation attitudes were almost equally correlated with Interest to learn, Fear (acceptance) and Knowledge. In contrast to comparison group, a low correlation between the “Interest to learn” and “Fear-acceptance” dimensions was found for the treatment group. Students who expressed less fear of bears were more interested in learning about this species. In both samples, Interest to learn correlated with Knowledge, but higher correlation was found in a treatment group. Much higher correlation than in treatment group was found between Fear (acceptance) and Knowledge in comparison group.

## 4. Discussion

This study indicates that gender is an important influencing factor concerning students’ attitudes to bears and bear topics. The findings are consistent with most previous research [30,45,46,47] and highlight the lower fear of bears (general influence on attitudes and detected as a differential result in the two subsamples) and more knowledge on bear topics (subsample 1) among male students compared to the female students and their higher proconservation attitudes and slightly greater interest to learn about this species (subsample 2). The greater fear among the females can be explained by perceived vulnerability [48]. These findings regarding willingness to learn about bears are in line with earlier research [49]. Still, the results are inconsistent with previous research results, except for girls’ somewhat greater interest to learn, compared to a study about wolves among Slovenian students [28]. This difference should, therefore, be considered in further research and while preparing educational activities.

The study showed no significant connection between place of residence and students’ attitudes to bears in general, in contrast to other studies among primary and secondary school children [23,32,50]. In subsample 2, the differential results revealed no differences in knowledge with respect to place of residence. Since every student in the subsample was given the same instruction, and therefore scored equally in the post-test, the findings were expected. Interestingly, the varying results (subsample 1) for the impact of living areas on students’ knowledge highlight the fact that rural students know more about bears than their counterparts in suburban and urban areas. Since such differences in knowledge in relation to place of residence were eliminated in subsample 2, the pre and post effect of workshops on the same students’ knowledge should be further explored.

Having seen a bear in nature also proved to be a key attitude-influencing factor in general, especially for reducing fear (both subsamples), and is consistent with previous research on wolves [28]. The research results also indicate that seeing a bear in nature was correlated with more knowledge of the species (subsample 1). Still, the connection was again not detected in subsample 2, presumably for similar reasons as mentioned above. More research is needed to understand this correlation better. Since the present findings show that seeing a bear in nature reduced fear, it is recommended that future research more systematically investigate how educational activities associated with observing bears in nature or outdoor learning about their habitats correlate with higher proconservation attitudes and behavior in nature in the long run. Some research already underscores the importance of direct experience for students forming a positive attitude [51] and the positive effect of exposure on bear habitat or bear presence in association with fear reduction [49] and greater support for conservation of the species [35].

Education level and enrolment in lower or upper secondary school influenced students’ attitudes to and knowledge of bears. The lower secondary school students in subsample 1 knew significantly less than their counterparts from upper secondary school and their attitudes were slightly less positive, but they showed a little more interest to learn about bear topics. In contrast, the students in subsample 2 enrolled in upper secondary school showed slightly lower proconservation attitudes than the younger counterparts in the lower secondary school program. Previous research among primary and secondary school children also revealed age-dependent attitudinal and knowledge scores [50].

Other factors, such as visiting a zoo, owning a dog, having a hunter in the family, and breeding livestock, had less impact on students’ attitudes and knowledge and need to be explored further. Students who regularly visit a zoo showed a somewhat greater interest to learn about bears, yet at the same time were a little more afraid of them. Since the effect was noticed only in this subsample, this aspect should be further investigated. Some previous studies e.g., [52] pointed to the importance of observing animals in a zoo setting, which can positively affect one’s attitudes. Still, irrespective of whether the observation of living animals occurs in a zoo setting or in nature, it arouses emotions and positively affects learning motivation [53,54]. Students in subsample 1 who reported having a hunter in the family were more knowledgeable than their counterparts. Since the sample was relatively small, more research to confirm this link is needed. Students from livestock breeding families also expressed slightly more knowledge of bears (subsample 1), although their attitudes were slightly less positive (subsample 2). Since the sample of livestock breeders was also small, further research is also needed to study this link. However, some connections with previous research on the local general public and stakeholder attitudes to bears revealed that most of the effort in communication and education should go towards younger livestock breeders [35]. The results also highlight the importance of owning a dog. Students in subsample 2 who were dog owners knew slightly more about bears than their counterparts. Since the effect was noticed only in subsample 2, the link between these two variables should be further explored. The research on the local general public established a link between dog-owning and greater tolerance with regard to human-bear conflicts with bears [35].

The study results also show that the educational treatment (the workshops on bear topics) contributed to significantly more knowledge of bears among the participating students. Thus, a significant finding of the research is that more knowledge about bears has an important effect on a higher interest to learn about bears, more proconservation attitudes, and partly reduced fear among students. A favorable effect of knowledge on students’ attitudes to carnivores was also established in earlier research [27,28,52]. A link between more knowledge of the species and higher proconservation attitudes has also been reported for the general public [35]. Since some differences between groups were detected in this study, more detailed research is needed to fully understand the interaction between knowledge and fear, and consequently human acceptance, as proposed by other authors, e.g., [55].

Expressed more fear by high achieving students within the treatment group can be linked to the indirect assessment of teaching. Using this approach for attitudes and knowledge assessment can be considered a limitation of this study. In future research, the influence of teaching should be directly observed with pre and post assessment of the same group of students, as was done for wolves [28]. On the other hand, the detected phenomenon within the treatment group, where students with the highest knowledge scores showed more fear than their counterparts, can be linked to the results of previous experimental research [20,56,57]. Knowledge was suggested as a strong moderator of cognitions on feelings for wolves, but not for brown bears [20]. Furthermore, previously mentioned research [56,57] showed that verbal information could either positively or negatively influence emotions in children. Since the brown bear species is fear-relevant, students with more newly gained knowledge of the species could become more aware of the possible dangers the species present. It has already been proposed [58] that education interventions should encompass information about the species and their habitats and the presentation of conflict avoidance strategies to lower the risk perceptions in humans. Other authors, e.g., [59,60], also reported that while such interventions correlate with greater knowledge and lower conflict avoidance behavior, these correlation with more positive attitudes were weak [59,61]. Therefore, future research regarding educational strategies that influence students’ knowledge and attitudes of brown bears should strongly rely upon socioscientific issues, as already proposed for wolves [27] and include information about the species’ benefits into the teaching activities [60]. In the future, it would be of importance to explore which knowledge dimension (especially procedural) students should develop, and which appropriate skills should be obtained that, when combined with positive attitudes, lead to proconservation behavior.

While predicting proconservation behavior, it is crucial to consider the psychological barriers that may prevent the development of proenvironmental behavior, even if one’s knowledge is great and attitudes positive. One of these inhibitory factors is perceived risk (e.g., physical or financial) [62]. For people who perceive the risk of a possible attack on family members or themselves, the constant feeling of fear can escalate to become an environmental stressor [63], which may further interfere with other everyday tasks [64], like avoiding outdoor-based activities [65]. In the long run, this kind of stress reduces life quality [66] and even policy support [67,68,69]. Various interventions possibly associated with reducing fear of large carnivore species have already been noted, such as information and education, direct exposure to animals or their habitats, participatory and collaborative approaches, and financial support [55]. Nevertheless, educational interventions are the most effective when they encompass direct experience [55,70,71,72,73].

## 5. Conclusions

Several findings of the present study are highlighted. A few sociodemographic factors (according to RQ1) significantly influenced the attitudes and knowledge of the students, such as gender or having seen a bear in nature. These factors should be considered in future research and while planning educational and communication activities. Other factors, such as residence, owning a dog, having a hunter in the family, breeding livestock, and visiting a zoo, had a smaller effect on the students’ attitudes and knowledge generally, meaning they should be explored further. The research also established differences between education levels (RQ 2), mainly regarding the students’ knowledge scores. The findings confirmed the assumption made in RQ3 that knowledge influences students’ attitudes to bears. A significant influence of knowledge on all attitude dimensions, i.e., interest to learn, conservation and fear, was shown. Further, the significant influence of instructions (workshops) was detected with respect to the students’ knowledge scores, but not regarding a change in attitude (associated with RQ4). Although, the present study indirectly explored the influence of the teaching (the workshops), a similar study should be performed on equivalent samples to directly investigate the influence of such teaching (pre and post testing of the same students).

Nevertheless, the study results show that more knowledge is correlated with stronger pro-conservation attitudes and partly with less fear. Through such workshops, students gain considerable knowledge about different species. Yet, it must be noted that, besides acquiring knowledge, a more effective attitudinal switch must be identified. Therefore, future conservation and management actions should employ education activities that include direct experiences with realia, or even observing live animals and their presence in their natural habitats. One possibility is observing live animals in the local zoos. Furthermore, teaching activities regarding large carnivore socioscientific issues should not be neglected, and scientific evaluation of the effectiveness of such actions should not be excluded.

## Figures and Tables

**Table 1:** Principal component analysis with an oblimin rotation of items to the individual attitudinal dimension.

Item	Component (PC)		
	I	II	III
**Conservation**			
Bears should be killed (exterminated). S_26_R *	0.743		
It would bother me if all the bears were culled in Slovenia. S_22	0.701		
In my opinion, there are too many bears in Slovenia. S_20_R *	0.677		
It is important to preserve brown bear species for future generations in Slovenia. S_10	0.662		
It is not necessary for the bears to be protected in Slovenia, as enough of them live elsewhere in Europe. S_06_R *	0.660		
Bears have the right to live and use forests, just like humans do. S_03	0.573		
**Interest to learn**			
I would like to learn about bears. S_09		−0.854	
I would also like to learn about species of bears that do not live in Slovenia. S_05		−0.809	
I want to know how bears feed, predate, and hibernate. S_21		−0.749	
I like to watch popular science shows about bears. S_01		−0.736	
I would like to learn about the damage caused by bears and conflicts with humans. S_15		−0.626	
**Fear—acceptance**			
I would be scared to walk in the woods if I knew bears were living there. S_23_R *			0.800
I am afraid of bears. S_04_R *			0.789
I would accept the presence of bears in the forests in my area without any problems. S_11			0.659
I would rather watch a movie about bears than see a living animal in the wild. S_27_R *			0.599
Kaiser-Meyer-Olkin (KMO)	0.826		
Bartlett’s test of sphericity	*χ2* = 2108.834 df = 105 *p* < 0.001		
Cronbach’s alpha	0.77	0.82	0.70
Eigenvalues	3.894	2.326	1.757
Explained variance (%)	25.96	15.50	11.72

* Reversed items. Principal component loadings over 0.40 are presented. Extraction Method: Principal Component Analysis. Rotation Method: Oblimin with Kaiser Normalization.

**Table 2:** GLM analysis of independent variables’ effect on the students’ attitude ratings.

Effect	Wilks’ L	F	Hypothesis df	Error df	*p*	Partial Eta Squared
Intercept	0.360	297.517	3	501	<0.001	0.640
Knowledge	0.868	25.496	3	501	<0.001	0.132
Upper/lower secondary	0.968	5.584	3	501	0.001	0.032
Gender	0.928	12.953	3	501	<0.001	0.072
Place of residence	0.981	1.572	6	1002	0.152	0.009
Seen in nature	0.955	7.906	3	501	<0.001	0.045

**Table 3:** Correlations between attitudinal dimensions and knowledge scores of the comparison group.

			Interest to Learn	Fear-Acceptance ***	Knowledge
**Spearman’s rho**	Conservation	Correlation coefficient	0.322 **	0.331 **	0.311 **
		Sig. (2-tailed)	<0.001	<0.001	<0.001
		*n*	317	317	317
	Interest to learn	Correlation coefficient		0.098	0.139 *
		Sig. (2-tailed)		0.081	0.013
		*n*		317	317
	Fear-acceptance	Correlation coefficient			0.398 **
		Sig. (2-tailed)			<0.001
		*n*			317

*** Correlations between fear-acceptance and other dimensions are positive due to reversed items. ** Correlation is significant at the 0.01 level (2-tailed). * Correlation is significant at the 0.05 level (2-tailed).

**Table 4:** Correlations between attitudinal dimensions and knowledge scores of the treatment group.

			Interest to Learn	Fear-Acceptance ***	Knowledge
**Spearman’s rho**	Conservation	Correlation coefficient	0.289 **	0.323 **	0.316 **
		Sig. (2-tailed)	<0.001	<0.001	<0.001
		*n*	217	217	217
	Interest to learn	Correlation coefficient		0.136 *	0.233 **
		Sig. (2-tailed)		0.045	0.001
		*n*		217	217
	Fear-acceptance ***	Correlation coefficient			0.202 **
		Sig. (2-tailed)			<0.001
		*n*			217

*** Correlations between fear-acceptance and other dimensions are positive due to reversed items. ** Correlation is significant at the 0.01 level (2-tailed). * Correlation is significant at the 0.05 level (2-tailed).

## Data Availability

The data presented in this study are available on request from the corresponding author.

## Appendix A

**Questionnaire: Used in the present study**

*Dear students. We invite you to take part in an anonymous survey (research) to assess your attitudes and knowledge about bears. Your cooperation will help us to improve pedagogical work and will influence the development and improvement of our nature conservation activities.*

	**Knowledge statements**	**Response**
1	Bears do not kill sheep and do not feed on them.	TRUE FALSE I DON’T KNOW
2	Brown bear is an endangered species in Slovenia.	TRUE FALSE I DON’T KNOW
3	The majority of brown bear’s diet represent the food of animal origin, such as roe deer.	TRUE FALSE I DON’T KNOW
4	By analysing the DNA from faeces samples, the bear kinship and abundance within certain areas could be assessed.	TRUE FALSE I DON’T KNOW
5	Beehives can attract bears and therefore lead to damage events.	TRUE FALSE I DON’T KNOW
6	By analysing DNA samples from bites on sheep, an individual bear who attacked or killed the animal can be determined.	TRUE FALSE I DON’T KNOW
7	When searching for fruits, such as plums, apples, and cherries, bears can cause damage to the fruit trees.	TRUE FALSE I DON’T KNOW
8	Brown bear represents a threat to humans.	TRUE FALSE I DON’T KNOW
9	Bears never kill the livestock and do not feed on them.	TRUE FALSE I DON’T KNOW
10	To protect people, brown bears do not need to be shoot.	TRUE FALSE I DON’T KNOW
11	Even if they have a strong odor, silage bales do not attract bears.	TRUE FALSE I DON’T KNOW
12	Brown bears walk around 7 km per day.	TRUE FALSE I DON’T KNOW
13	Bears feed on corn at the cornfields.	TRUE FALSE I DON’T KNOW
14	The cull is not a suitable management approach for damage prevention on small livestock.	TRUE FALSE I DON’T KNOW
15	Waste in the bins outside the houses can attract bears.	TRUE FALSE I DON’T KNOW
16	In nature, brown bears predate the healthiest herbivores.	TRUE FALSE I DON’T KNOW
17	In Slovenia, the brown bear is an alien species.	TRUE FALSE I DON’T KNOW
18	Bears usually visit sites with unprotected waste just once.	TRUE FALSE I DON’T KNOW
19	Brown bear is a solitary animal.	TRUE FALSE I DON’T KNOW
20	Dogs, even if properly trained, cannot protect livestock from predation.	TRUE FALSE I DON’T KNOW
21	Humans can replace the ecological role of brown bears in nature.	TRUE FALSE I DON’T KNOW
22	An electric fence is an effective measure for deterring bears from beehives.	TRUE FALSE I DON’T KNOW
23	The majority of brown bear cubs die before reaching the age of 1 year.	TRUE FALSE I DON’T KNOW
24	On the small pasture, livestock can be protected from bear attacks by an electric fence.	TRUE FALSE I DON’T KNOW
25	Overpopulation of bears in Slovenia causes attacks on livestock.	TRUE FALSE I DON’T KNOW
26	Waste bins should be put in front of the houses in the morning, right before the collection. In that case, bears do not have time to access them.	TRUE FALSE I DON’T KNOW

**The meaning of the scale:****1** - I completely disagree, **2** - I disagree, **3** - indefinite, **4** - I agree, **5** - I completely agree

	**Attitude items**	**Value**
S_01	I like watching popular science shows about bears.	1 2 3 4 5
S_03	Bears have the right to live and use forests, just like humans.	1 2 3 4 5
S_04_R	I am afraid of bears.	1 2 3 4 5
S_05	I would like to learn about bear species that do not live in Slovenia.	1 2 3 4 5
S_06_R	In Slovenia, it is not necessary for bears to be protected, as there are enough of them living elsewhere in Europe.	1 2 3 4 5
S_09	I would like to learn about bears.	1 2 3 4 5
S_10	It is important to preserve the brown bear species for future generations.	1 2 3 4 5
S_11	I would accept the presence of bears in the near woods without difficulty.	1 2 3 4 5
S_15	I would like to learn about the damage caused by bears and about conflicts with humans.	1 2 3 4 5
S_20_R	I think that there are too many bears living in Slovenia.	1 2 3 4 5
S_21	I would like to know how bears feed, hunt, and hibernate.	1 2 3 4 5
S_22	It would bother me if all the bears in Slovenia were killed.	1 2 3 4 5
S_23_R	I would be afraid of walking in the woods if I knew bears live there.	1 2 3 4 5
S_26_R	It would be best to exterminate (kill) all bears.	1 2 3 4 5
S_27_R	I would rather watch a movie about a bear than see a live animal in the wild.	1 2 3 4 5

**For the purposes of statistical data analysis, please fill in all sections below**.

**Gender:** male | female	**Age:** __________
**Grade** || **Study year:** | 6 | 7 | 8 | 9 || 1 | 2 | 3	
**I live in/at:** [ ] the city | [ ] the suburbs | [ ] the countryside	
**I visit nature:** [ ] (Almost) everyday | [ ] Once a week | [ ] Once a month | [ ] Occasionally	
**I visit ZOO:** [ ] less than once per year | [ ] 1–3 times per year	
**Place of residence**: _______________________	
**I have a hunter in my family** yes | no	
**In my family, we breed livestock.** yes | no	
**I have already encountered a bear in nature.** yes | no	
**I have/had a dog as a pet.** yes | no	
